# Point-of-Care Ultrasound in the Assessment of Gastric Residual Volume: Protocol for a Scoping Review

**DOI:** 10.2196/84080

**Published:** 2026-04-27

**Authors:** Fernanda Raphael Escobar Gimenes, Renata Pinheiro Lopes, Mariana de Magalhaes Framartino, Rosana Aparecida Pereira, Priscilla Roberta Silva Rocha, Vinicius Batista Santos

**Affiliations:** 1 Department of General and Specialized Nursing, University of São Paulo at Ribeirão Preto College of Nursing, Avenida Bandeirantes, 3900, Ribeirão Preto, 14040-902, Brazil, 55 11 3091-3232; 2 Department of Nursing, University of Brasília, Faculty of Health Sciences and Technology, Brasilia, Brazil; 3 Department of Clinical and Surgical Nursing, Federal University of São Paulo, Paulista School of Nursing, São Paulo, Brazil

**Keywords:** point-of-care ultrasonography, gastric residual volume, scoping review, pulmonary aspiration, I-AIM framework

## Abstract

**Background:**

Pulmonary aspiration of gastric contents is a serious clinical complication, particularly in patients receiving enteral nutrition or undergoing anesthesia. Gastric residual volume (GRV) is a commonly used surrogate marker that can identify delayed gastric emptying and enteral nutrition intolerance, both of which may increase the risk of aspiration. Traditional methods for measuring GRV are invasive and lack standardization. Point-of-care ultrasound (PoCUS) has emerged as a promising, noninvasive bedside alternative. Despite its growing clinical use, there is still no consolidated guidance on PoCUS procedures for GRV assessment in adult patients.

**Objective:**

This scoping review aims to map and synthesize the available evidence on procedural techniques, interpretation criteria, and decision-making applications related to PoCUS for GRV assessment.

**Methods:**

This scoping review will follow the JBI Collaboration methodology and will be reported in accordance with the PRISMA-ScR (Preferred Reporting Items for Systematic Reviews and Meta-Analyses Protocols extension for Scoping Reviews) guidelines. The PCC (population, concept, and context) mnemonic guided the formulation of the research question. Systematic searches will be conducted in MEDLINE, CINAHL, Embase, Scopus, Cochrane Library, and LILACS, as well as in gray literature sources. Eligible sources will include primary studies, reviews, and clinical guidelines focused on PoCUS for GRV assessment in adults (aged ≥18 years). Two independent reviewers will perform study screening and data extraction. The synthesis will be structured using the I-AIM (indication, acquisition, interpretation, and medical decision-making) framework. Results will be summarized narratively, in tables, and through visual representations such as flowcharts and conceptual diagrams.

**Results:**

This protocol was registered on the Open Science Framework registry on August 21, 2025. The literature search began on April 3, 2026, with study selection and data extraction planned for May 2026. The final results are expected to be submitted for publication in July 2026. As this is a protocol study, no results are available yet.

**Conclusions:**

This review will identify and categorize the procedures, technical parameters, and clinical applications of PoCUS for GRV assessment in adult patients. By mapping the existing evidence, the findings may inform future research, educational curricula, and the development of clinical guidelines for nurses. In addition, the review will highlight methodological gaps and variations in practice that may affect the safe and effective use of PoCUS across diverse health care settings.

## Introduction

Pulmonary aspiration, defined as the entry of oropharyngeal or gastric contents into the lower airways [1], is a serious complication that can lead to aspiration pneumonia and other potentially fatal conditions [2-4]. Older male patients, those with enteral feeding tubes, and those with neurological, gastrointestinal, or pulmonary disorders are at increased risk for this event [4,5]. In anesthetic settings, aspiration of gastric contents is associated with high mortality and permanent injury, particularly when it results from failures in implementing safe practices [6].

Previous studies have reported an aspiration incidence of 2 to 7 per 20,000 anesthetic procedures, reaching 0.5% in emergency surgeries performed in operating rooms, and rising to 2.7% in emergency procedures performed outside surgical settings [7]. Among patients receiving enteral nutrition, aspiration pneumonia is also common, with prevalence ranging from 4% to 95% and mortality rates from 17% to 62% [5].

One of the main risk factors for aspiration is elevated gastric residual volume (GRV), which indicates impaired gastrointestinal motility, enteral nutrition intolerance, and delayed gastric emptying [5,8]. These conditions contribute to reflux and vomiting, thereby increasing the risk of pulmonary aspiration, especially in patients with a BMI greater than 30 kg/m^2^ and in those with type 2 diabetes mellitus undergoing anesthetic induction [5]. Traditional GRV measurement involves active aspiration with a syringe or passive drainage by gravity. However, these techniques are limited by variability in results and a lack of standardization [8,9].

In this context, point-of-care ultrasound (PoCUS) has emerged as a promising alternative for GRV assessment. PoCUS refers to the use of portable ultrasound performed directly by health care professionals at the bedside for real-time diagnostic or monitoring purposes. It is an objective, focused approach integrated into clinical reasoning that may support faster and safer decision-making in acute and dynamic settings [10].

**Figure 1.**
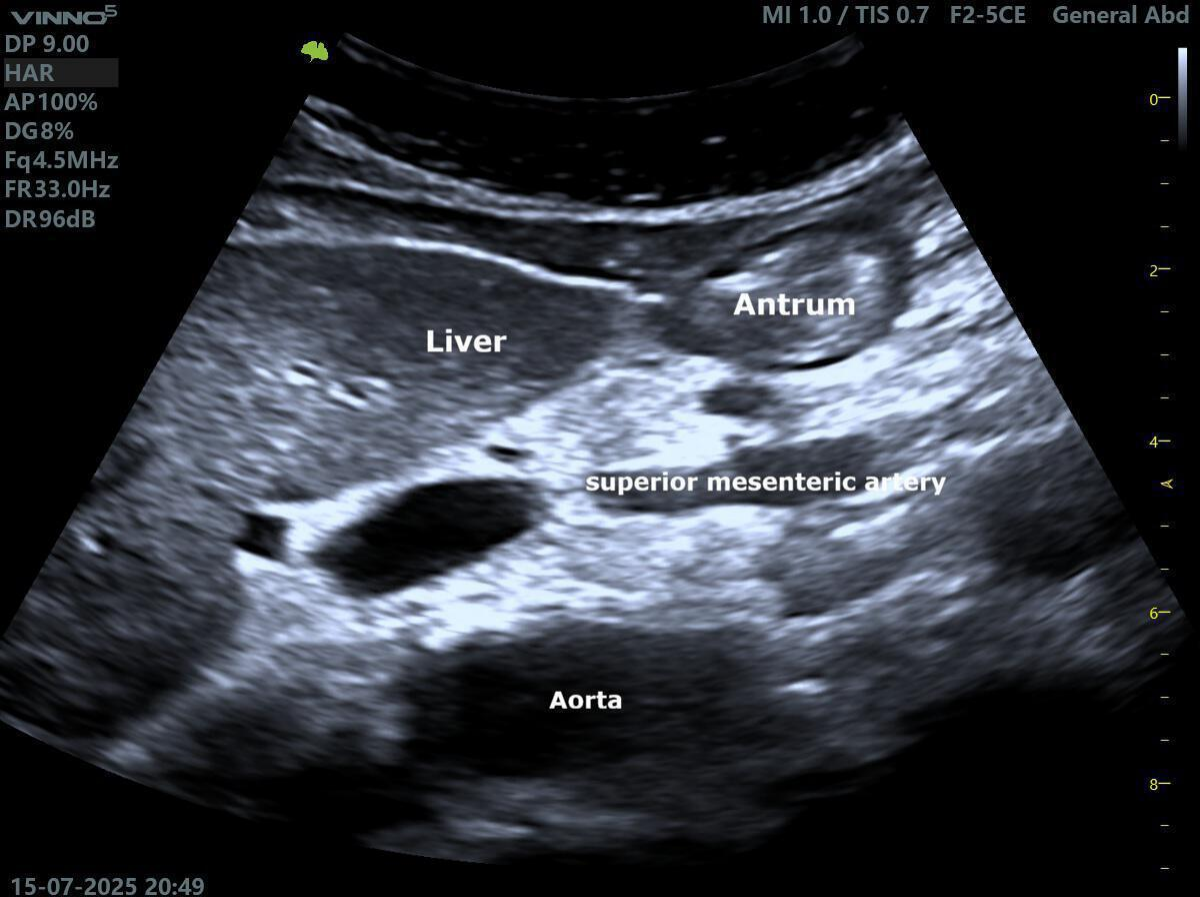
Point-of-care ultrasound (PoCUS) illustration of the gastric antrum.

For GRV estimation, PoCUS enables assessment of gastric contents by visualizing the gastric antrum in the transverse plane, typically with the patient in the right lateral decubitus position. This structure (Figure 1), located between the left lobe of the liver and the aorta or superior mesenteric artery, can be measured and converted into an estimated volume using validated formulas, such as that proposed by Perlas [11], providing a noninvasive and effective alternative to traditional methods. In addition, this approach may support aspiration risk stratification and improve the safety of enteral nutrition and preoperative fasting [5,8,11,12].

A recent study showed that ultrasound examinations performed by nurses in intensive care units for GRV assessment and verification of nasogastric tube position correlated strongly with conventional methods, and were safe, simple, and effective [13]. Although GRV thresholds commonly used to interrupt enteral nutrition range from 200 to 250 mL [14], current American Society for Parenteral and Enteral Nutrition guidelines recommend interrupting enteral nutrition in critically ill patients only when GRV exceeds 500 mL in the absence of other signs of intolerance [15]. Similarly, the European Society for Clinical Nutrition and Metabolism (ESPEN) recommends delaying enteral nutrition in patients with GRV greater than 500 mL, without distinguishing among patient populations [16].

Despite advances in the clinical use of PoCUS, important gaps remain in the literature regarding the specific techniques used to assess GRV in adults. A preliminary search of PROSPERO, OSF, Web of Science, and the Cochrane Library identified no review protocols on this topic. Moreover, no consolidated guidelines or standardized protocols currently support the safe and effective use of PoCUS by nurses for this purpose. Therefore, this scoping review is proposed to map and synthesize the available evidence on procedures used to assess GRV with PoCUS in adult patients. Specifically, the review aims to identify the clinical indications, image acquisition techniques, sonographic interpretation methods, and medical decision-making application related to GRV estimation using PoCUS across diverse clinical settings. Secondary objectives are as follows:

- To classify the reported procedural techniques according to the I-AIM framework (indication, acquisition, interpretation, and medical decision-making) framework [17].
- To identify gaps in the literature regarding standardization and training for PoCUS-based GRV assessment.
- To inform future guidelines and educational strategies for the safe and effective implementation of PoCUS in nursing and other health care professions.

## Methods

### Protocol and Registration

This scoping review will be conducted in accordance with JBI Collaboration methodology [18] and reported in accordance with the PRISMA-ScR (Preferred Reporting Items for Systematic Reviews and Meta-Analyses Extension for Scoping Reviews) guidelines [19].

The review protocol was registered on the OSF platform [20].

### Research Question

The Population, Concept, and Context (PCC) mnemonic was used to formulate the research question. Within this framework, the Population (P) comprised adults and older adults (aged ≥18 years), the Concept (C) involves assessment of GRV using PoCUS, and the Context (C) included the various clinical settings in which PoCUS is used for this purpose. Accordingly, the central question guiding this scoping review is as follows: What procedures are used when applying PoCUS to assess gastric residual volume in adult patients across clinical care settings?

### Eligibility Criteria

Eligible sources will include primary studies, reviews, and clinical guidelines focused on the use of PoCUS for GRV assessment in adult patients (aged ≥18 y). Exclusion criteria will include: (1) studies involving children or animals; (2) abstracts, letters, or expert opinion; (3) protocols and trial registrations; and (4) studies not focused on the stomach (eg, ultrasound of other organs). No date or language restrictions will be applied.

### Information Sources

Systematic searches will be conducted in the following databases: PubMed (via National Institutes of Health), CINAHL (via EBSCOhost), Embase (via Elsevier), Scopus (via Elsevier), the Cochrane Library (via Wiley), LILACS (via the Virtual Health Library, in Portuguese, Biblioteca Virtual de Saúde), and Web of Science Core Collection (via Clarivate). Gray literature will be searched through OpenAlex, ProQuest Dissertations & Theses Global (via Clarivate), and institutional websites. To ensure comprehensive identification of relevant evidence, additional search strategies will also be used. Specifically, backward citation tracking will be conducted by manually screening the reference lists of all included studies, reviews, and guidelines to identify additional eligible sources that may not have been retrieved in the initial database search.

### Search Strategy

The search strategy was developed to address the research question using the PCC framework by combining MeSH (Medical Subject Headings) terms and Health Sciences Descriptors in Portuguese (Descritores em Ciências da Saúde) descriptors with relevant keywords and synonyms (Table 1 and Multimedia Appendix 1). Terms were refined using Boolean operators. The initial search was conducted in PubMed.

**Table 1.** Population, concept, and context (PCC) framework applied to the research question and search terms.

PCC elements	Description	Search terms used
Population	Adults and older adults (aged ≥18 years)	(“Adult”[MeSH Terms] AND “Aged”[MeSH Terms])
Concept	Assessment of GRV^a^ using PoCUS^b^	(Ultrasound OR Ultrasonography OR “Diagnostic Ultrasounds” OR “Ultrasound Imaging” OR “Bedside Test” OR “Bedside Testing” OR “Bedside Technology” OR “Bedside Technologies” OR “Point of Care” OR “Point-of-Care” OR “Point of Care Systems” OR “Point-of-Care Systems” OR “Point-of-Care System” OR “Point of Care System” OR “Point of Care Technology” OR “Point-of-Care Technology” OR “Point of Care Testing” OR POCUS OR “Point-of-care ultrasonography” OR “Point of care ultrasonography” OR “Point-Of-Care Ultrasound” OR “Point Of Care Ultrasound” OR Sonography OR Ultrasonic* OR ultrasso* AND “Gastrointestinal Contents” OR “Gastric Residual Volume” OR “residual gastric volume” OR “gastric volume” OR “gastric content” OR “Gastric residuals” OR antrum OR “antral area”) AND (assess* OR Evaluation OR avalia*)
Context	Any clinical settings where PoCUS is used for GRV assessment	Perioperative care, intensive care, emergency, anesthesia, critical care, and others.

a GRV: gastric residual volume.

b PoCUS: point-of-care ultrasound.

A search validation procedure was also used. A set of known key studies relevant to the use of PoCUS for GRV assessment in adult patients was identified in advance by the review team based on prior knowledge and preliminary exploration of the literature (Multimedia Appendix 2 [11,21,22]). These studies served as benchmark references to validate the comprehensiveness and sensitivity of the search strategy. The initial search was tested to ensure the retrieval of these benchmark studies. If any benchmark studies were not retrieved, the search strategy was refined accordingly by adjusting keywords, controlled vocabulary (eg, MeSH terms and Descritores em Ciências da Saúde), and Boolean operators until all key studies were successfully captured.

For the purposes of this scoping review, PoCUS was defined as the use of portable ultrasound performed directly by a health care provider at the point of care, for real-time diagnostic or clinical monitoring purposes [23]. Gastric residual volume is widely recognized as a surrogate parameter for identifying gastrointestinal motility disorders, particularly delayed gastric emptying. Its measurement has traditionally been used in clinical practice as an indirect indicator of enteral nutrition intolerance and increased risk of complications such as pulmonary aspiration [24].

### Screening and Selection Process of Included Studies

Search results will be imported into Rayyan for deduplication and blinded screening. Two reviewers will independently screen titles and abstracts, followed by full-text screening, and any disagreement will be resolved by a third reviewer. Reasons for full-text exclusion will be recorded and reported in a PRISMA-ScR flow diagram [19].

Study authors will be contacted when clarification is needed regarding study eligibility, missing or unclear methodological details, or incomplete data relevant to the review objectives. Initial contact will be made by email using a standardized message template adapted from the OSF repository [25]. Authors will be given two weeks to respond. If no reply is received, a single follow-up message will be sent.

Consultation with content experts in clinical nutrition, PoCUS, and nursing will be undertaken to identify unpublished data, practice guidelines, or ongoing research that may contribute to the review will also be performed.

### Data Items and Extraction Process

A customized data charting form, adapted from the JBI template [26], will be used. Two reviewers will independently extract data, followed by reconciliation. Pilot extraction will be conducted on a sample of studies to refine the form and ensure consistency. Extracted data will include the following:

- Bibliographic information; title, authors, year, and country
- Study characteristics; design, setting, sample, and operator profession
- Alignment of PoCUS procedures with I-AIM [17], including
  - indication (clinical context for GRV assessment)
  - acquisition (patient positioning, transducer type, scanning windows, and ultrasound settings)
  - interpretation (sonographic markers, grading scales, and formulas)
  - medical decision-making (use of findings to adjust care, eg, feeding, fasting, and aspiration risk)
- Other information; training protocols, facilitators or barriers, and guideline recommendations

Incomplete or partially reported data will not be used as exclusion criteria. Data will be extracted as reported, and missing information will be coded as “not reported” in the extraction tables. No imputation will be performed. The extent and patterns of missing reporting will be described narratively and summarized in tables to enhance transparency and allow readers to interpret the evidence map appropriately.

### Data Synthesis

The analysis will include both quantitative descriptive summaries and qualitative synthesis. Quantitative data will be summarized using descriptive statistics, including frequencies, proportions, ranges, and measures of central tendency when applicable. Because of substantial heterogeneity in study designs, outcome measures, and reporting formats, no meta-analysis is planned. Heterogeneous measures (eg, different GRV thresholds, measurement techniques, and patient positioning) will be grouped and presented in structured tables to allow comparison across studies.

For the qualitative synthesis, a thematic analysis approach will be used. Extracted data will be coded inductively and organized into categories aligned with the I-AIM framework domains: indication, acquisition, interpretation, and medical decision-making. Two reviewers will independently code the extracted data. Disagreements will be resolved through discussion, and when consensus cannot be reached, a third reviewer will be consulted.

The I-AIM framework will be operationalized as an analytic matrix during both data extraction and synthesis. Each included study will be mapped according to the following:

- Indication; clinical contexts and purposes for GRV assessment using PoCUS
- Acquisition; technical procedures, equipment, patient positioning, and operator-related aspects
- Interpretation; criteria used to estimate GRV and define intolerance risk
- Medical decision-making: how PoCUS findings informed clinical management

Findings will be synthesized narratively and presented in tables and visual diagrams to identify patterns, variations, and evidence gaps across studies.

No risk-of-bias appraisal and no meta-analysis are planned, in line with JBI methodological guidance for scoping reviews. The findings will therefore be synthesized using narrative description and structured tabular presentation only.

## Results

This scoping review protocol was registered in the OSF on August 21, 2025, and received funding on August 8, 2025 (process number 305617/2024‐9), and February 6, 2026 (process number 2025/21327‐5). The literature search began on April 3, 2026. Screening and data extraction will occur in May 2026. Data analysis has not yet begun, and the synthesis of results is expected to be finalized by June 2026. The final manuscript is expected to be submitted for publication in July 2026.

## Discussion

### Overview

This protocol outlines a structured approach to systematically map how PoCUS has been used to assess GRV in adult patients. Its anticipated main contribution is the identification and organization of procedural techniques, interpretation criteria, and clinical decision-making applications across different care settings. Although results are not yet available, this review is expected to provide an integrated overview of how PoCUS for GRV assessment is currently described and operationalized in the literature, while also highlighting areas of variability, gaps, and emerging practices.

### Principal Expected Contributions

The primary anticipated contribution of this scoping review is the development of a comprehensive map of how PoCUS has been applied to assess GRV, structured according to the I-AIM framework domains: indication, acquisition, interpretation, and medical decision-making [11]. By organizing the evidence in this way, the review is expected to clarify how technical procedures are reported, which clinical contexts most commonly support their use, and how findings are incorporated into care decisions such as enteral nutrition management and aspiration risk assessment. This structured synthesis may help to reduce conceptual fragmentation and provide a clearer reference for clinical and research.

### Comparison With Existing Knowledge

Existing literature suggests growing interest in PoCUS as a noninvasive bedside method for evaluating gastric contents and supporting clinical decision-making. However, descriptions of procedural details, measurement approaches, and interpretation thresholds appear to be dispersed across disciplines and clinical contexts [12,27,28]. The anticipated findings of this review are expected to extend previous narrative and empirical work by systematically compiling how these procedures are operationalized, rather than focusing exclusively on effectiveness. This may help identify inconsistencies in terminology, heterogeneity in acquisition techniques, and differences in how PoCUS findings are translated into clinical actions.

### Strengths and Limitations

This protocol has several methodological strengths. It follows JBI methodology for scoping reviews, applies the PRISMA-ScR reporting framework, and includes a comprehensive search strategy across multiple databases and gray literature sources. The use of the I-AIM framework [11] as an analytic structure is expected to support a consistent, theory-informed synthesis of heterogeneous data.

### Future Directions and Implications

By identifying gaps in procedural reporting, training approaches, and clinical integration, this review is expected to inform future research priorities, including the need for standardized protocols and competency frameworks. The findings may also support the development of educational strategies and guide future validation studies focused on reliability, implementation, and clinical outcomes associated with PoCUS-based GRV assessment.

### Dissemination Plan

The results of this review will be disseminated through peer-reviewed publications, conference presentations, and the sharing of extracted datasets and synthesis materials via the OSF platform. These strategies aim to promote transparency, facilitate knowledge translation, and support the incorporation of evidence into clinical education and practice, particularly in multidisciplinary and nursing-led care settings.

### Conclusions

This protocol describes a systematic and transparent approach to mapping the available evidence on the use of PoCUS for GRV assessment in adult patients. By organizing the literature according to procedural domains and clinical applications, the planned review aims to clarify how this technique is currently used, identify areas of variation, and highlight gaps that may guide future research and practice development. As a scoping review protocol, the study does not seek to determine effectiveness but rather to provide a structured overview that may support future standardization efforts, training initiatives, and evidence-informed integration of PoCUS into clinical care.

## Supplementary material

10.2196/84080 Multimedia Appendix 1 Search strategy and results.

10.2196/84080 Multimedia Appendix 2 Key papers.

## Abbreviations

GRV: gastric residual volume
I-AIM: indication, acquisition, interpretation, and medical decision-making
MeSH: Medical Subject Headings
PCC: population, concept, and context
PoCUS: point-of-care ultrasound
PRISMA-ScR: Preferred Reporting Items for Systematic Reviews and Meta-Analyses extension for Scoping Reviews
