# Supplementary material for: Point-of-Care Ultrasound in the Assessment of Gastric Residual Volume: Protocol for a Scoping Review
Source: JMIR Res Protoc. 2026 Apr 27;15:e84080. doi: 10.2196/84080 (PMC13121960; doi:10.2196/84080)
Supplement: Multimedia Appendix 1 [file resprot-v15-e84080-s001.docx]

**Key Papers:**

11. Perlas A, Mitsakakis N, Liu L, Cino M, Haldipur N, Davis L, Cubillos J, Chan V. Validation of a mathematical model for ultrasound assessment of gastric volume by gastroscopic examination. Anesth Analg. 2013 Feb;116(2):357-63. doi: 10.1213/ANE.0b013e318274fc19.

12. Perlas A, Chan VWS, Lupu CM, Mitsakakis N, Hanbidge A. Ultrasound assessment of gastric content and volume. Anesthesiology. 2009;111:82-9.

13. Perlas A, Davis L, Khan M, Mitsakakis N, Chan VWS. Gastric sonography in the fasted surgical patient: a prospective descriptive study. Anesth Analg. 2011;113:93-7.
